# For-Profit Nursing Homes in the Netherlands: What Factors Explain Their Rise?

**DOI:** 10.1177/0020731420915658

**Published:** 2020-04-10

**Authors:** Aline Bos, Florien Margareth Kruse, Patrick Paulus Theodoor Jeurissen

**Affiliations:** 1 Utrecht University School of Governance, Utrecht, the Netherlands; 2 Scientific Center for Quality of Healthcare (IQ Healthcare), Radboud University Medical Center, Nijmegen, the Netherlands *Both the authors share first authorship of this article.

**Keywords:** for-profit, nursing homes, nonprofit, private equity, ownership, the Netherlands

## Abstract

This exploratory, mixed-methods study analyzes characteristics of the emerging for-profit nursing home industry in the Netherlands and identifies the interrelated set of factors (context, trends, and sector conditions) that contribute to its growth. Until recently, the Dutch nursing home sector relied almost exclusively on nonprofit providers. Even though profit distribution in nursing home care is still banned, the for-profit nursing home sector is expanding. The study uses economic theory on nonprofit organizations and mixed-form markets to understand this expansion. We find that changes in the regulatory framework have unlocked the potential of the for-profit nursing home sector, enabling for-profit nursing homes to circumvent the for-profit ban. The expansion of the for-profit sector was mainly driven by the low responsiveness of the nonprofit sector to increased and changed demands. For-profit providers took advantage of this void. Moreover, they exploited “cream-skimming” potential in the market and used the wider care system to reduce their labor costs by relying on external specialist care. Another main driver was the access to financial capital from private investors (e.g., private equity firms).

Nursing homes can be public, nonprofit, or for-profit organizations. The share of for-profit nursing homes differs significantly among Western countries, ranging from 4% in Norway to about 76% in England.^1^ For-profit nursing homes have received considerable attention from scholars, mainly with regard to their performance in comparison to nonprofit and public organizations.^2–7^ Research on factors that explain the role of for-profit organizations in the nursing home industry is less advanced. Although literature on the nonprofit enterprise offers helpful insights about factors that might shape the organizational makeup of sectors, scholars also state that “there is very little understanding of the dynamic forces causing the expansion of the [nonprofit or for-profit] sector into areas long dominated by the other.”^8(p544),9(p63)^

Current developments in the Dutch nursing home sector provide a good opportunity to increase our understanding of these dynamics. The Netherlands is known for its almost exclusively private, nonprofit provision of nursing home care.^10^ Until recently, the role of for-profit providers was negligible. No Dutch policies were directed toward the growth of the for-profit share, and a ban on profit distribution in nursing home care for the elderly is still in place. Nevertheless, Dutch for-profit nursing homes are gaining ground.

This explorative study aims to understand how the Dutch nursing home market has opened up to for-profit homes: *What is the current status of the Dutch for-profit nursing home sector, and what factors stimulated its expansion?* It is, to the best of our knowledge, the first academic study aimed at describing and understanding the growth in for-profit nursing homes in the Netherlands. Our study builds on mixed-form markets literature^11,12^ and economic theory on nonprofit organizations.^13–17^

## Theoretical Framework

### For-Profit and Nonprofit Organizations

The principal difference between for-profit and nonprofit organizations is “the presence of strict limits on the appropriation of the organization’s surplus in the form of monetary gain by those who run and control it.”^18(p5)^ Both nonprofit and for-profit organizations can earn a surplus, but the non-distribution constraint prohibits nonprofit organizations from *distributing* surpluses to third parties. Instead, they must retain and devote surpluses to financing further development of their services, to benefit “beneficiary stakeholders.”^14,16^

In order to understand the participation of for-profit providers in the health care system, it is useful first to review theories explaining the participation of nonprofit providers. The “third-sector rationale” and the “contracting and trust-goods rationale” help to explain the presence of nonprofit organizations in certain industries.^11^ The “third-sector rationale” understands the participation of nonprofit organizations in a sector as a way of compensating for inadequate for-profit and government provision of services.^18,19^ Nonprofit providers might seek to step in, for example, when profit-maximizing behavior by for-profit providers, such as cost-cutting, leads to a reduction in the quality of services or when government providers are unable to deal with heterogeneity in demand.^17^ The “contracting and trust-goods rationale” views the organization instead as a nexus of contracts: It argues that, rather than a corrective for the failures of other providers, nonprofit providers are the most efficient form of organizing the delivery of “trust goods” – that is, goods that are difficult for stakeholders to evaluate due to information asymmetry. Because nonprofit providers are subject to the non-distribution constraint, consumers are less concerned about being exploited due to the information asymmetry, and hence the costs of contracting are lower, because less effort must be made to regulate and control the contracted providers.^11,13,14,17,19^

### Factors That Stimulate the Entrance of For-Profit Organizations in Nonprofit Sectors

The aforementioned theoretical arguments predict that the nonprofit sector dominates in the provision of long-term care (LTC) services; however, many Western health care systems are organized as mixed markets that also include for-profit organizations.^1^ The Dutch nonprofit nursing home sector is also evolving into a mixed market. Literature on mixed-form markets points to possible reasons for the coexistence of different organizational forms in one sector^12,20^ and helps us to identify factors that might explain the changing makeup of the Dutch nursing home sector. We identify sector conditions, broader trends, and context enablers.

#### Sector conditions

The profit motive incentivizes for-profit firms to enter a sector and expand when demand increases or changes.^14^ In addition, for-profit organizations are more responsive to changing demand than nonprofit providers because they do not face “trapped capital.”^21^ Although nonprofit organizations aim at avoiding a negative net cash flow, they are not necessarily incentivized to minimize costs and to adjust capacity to demand. Hence, nonprofit organizations tend to be slower in adjusting their capacity to changing demands than for-profit organizations.

A related factor that might lead to an increase of for-profit providers is heterogeneity in demand, which gives nonprofit and for-profit organizations the opportunity to serve their own clientele. For example, nonprofit nursing homes in the United States primarily target the “clinically more severe and financially more lucrative end of the payer spectrum,” whereas for-profit facilities “usually have a less lucrative payer mix.”^22(p339)^

A related condition is the potential for “cream skimming.” It is not unusual for nonprofit organizations to cross-subsidize their services.^14^ The surplus of payments made by individual clients is used to serve nonprofit organizations’ charitable clients. As for-profit organizations can choose to serve only profitable clients, they are able to compete on price and/or quality of services.^11,12^ In general, increasing prices in nonprofit organizations beyond a break-even point signals the market’s potential profitability, which may lead to for-profit organizations entering the market.^20^

#### Broader trends

Sector conditions are affected by broader trends: demographic developments, labor market circumstances, financial trends, and technological developments.^19^ For example, an aging population would lead to an increase in demand for LTC services. Labor market circumstances determine the type of labor available and the fluctuations in labor costs. The nonprofit and for-profit sectors may attract different types of labor and therefore changing labor market circumstances may affect them differently. For instance, the nonprofit sector attracts more voluntary labor, so rising labor costs may give nonprofit organizations a competitive advantage over for-profit organizations.^16^ Trends in the cost of financial capital can also affect the ownership composition. Nonprofit and for-profit organization exploit different ways of attracting investment funds. For-profit organizations are able to attract private investors, such as private equity firms, because they can pay dividends, whereas nonprofit organizations rely on financial means such as loans, donations, or grants. Finally, technological developments can lead to innovations that disrupt the composition of the market.^23^

#### Context

**Figure 1.**
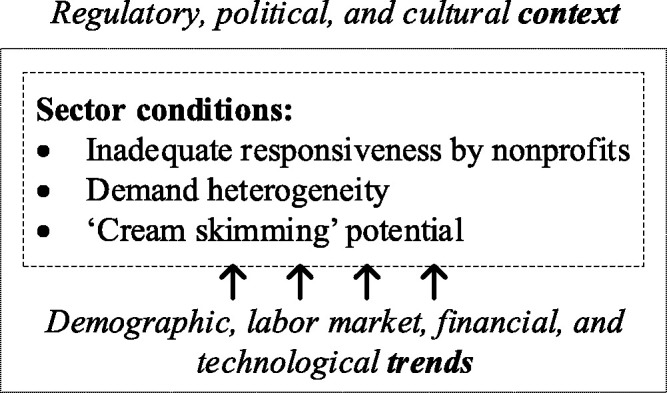
Summary of factors that can facilitate for-profit entry in nonprofit-dominated sectors.

These conditions and trends need to be placed in their regulatory, political, and cultural contexts.^19^ Several contextual factors affect the emergence and growth of the for-profit sector. First, regulations can either promote or hinder the role of for-profit organizations.^11^ For example, government regulations granting tax-exemptions to nonprofit organizations give them a competitive advantage over for-profit providers. Second, the political and cultural context can be either receptive to or skeptical of for-profit provision of health care services. For example, different types of welfare states can lead to different approaches to problem-solving that favor one organizational form over the other because of more or less trust in the private sector. The American liberal welfare state favors for-profit provision, whereas the social-democratic welfare states in Scandinavia favor public provision.^24^ Third, path dependencies affect the emergence of for-profit providers: The “social origins” of public goods provision and existing institutions create structures, norms, and practices that can significantly influence the organizational makeup of the sectors.^19,24^ Fourth, the political and cultural context can be subject to broad, paradigmatic shifts. Most notably, the New Public Management paradigm of the 1980s and 1990s encouraged business-like values such as efficiency, output measurement, and customer orientation.^25^ New Public Management heralded an era of privatization, tendering procedures for public services, and outsourcing. In many countries, the for-profit nursing home sector grew in response to the introduction of quasi-markets.^6,26–30^ Figure 1 shows the schematic representation of the theoretical framework.

## Institutional Background

The comprehensive, universal LTC system in the Netherlands enables every citizen in need of LTC to rely on public funding. The Netherlands is one of the highest LTC spenders on nursing and personal care services among all Organisation for Economic Co-operation and Development countries.^31^

In 2015, a major reform of LTC regulation in the Netherlands occurred. The reform aimed to bring about a move from residential to non-residential care.^32^ It also decentralized the LTC sector, delegating commissioning power to regional LTC offices. The reform reduced government responsibility: Instead of having overall control of LTC delivery, the government would instead finance and safeguard the functioning of the LTC market.

For a person to get access to LTC and public financing in the Netherlands, they must undergo both a care needs assessment and means testing. The care need is determined by the Care Assessment Centre and gives a person access to public LTC funds (Wlz; Dutch LTC law). The Wlz regulation provides 3 options for care financing. The first and most frequently chosen option is the in-kind intramural package, which is used in nonprofit nursing homes. It is an elaborate care package that includes housing. For the in-kind intramural package, a regional LTC office contracts nursing homes within its region. People choosing the in-kind package are placed in a contracted LTC facility based on the nursing home’s suitability and vacancies. The second financing plan is an in-kind extramural package called the total home-care package (HCP; in Dutch: VPT) or the modular care package (MCP; in Dutch: MPT). In this financing plan, the regional LTC office only purchases the provision of care; care recipients organize and finance their own housing. This can be their own house or an apartment on the site of a nursing home. With MCP, the care is still contracted by the regional LTC office, but the eligible person can adapt the care package – for example, by abstaining from food services in the HCP package. The third option is funding in the form of a personal budget (PB; in Dutch: PGB), which allows clients to arrange their own extramural care instead of delegating this task to the regional LTC office. As both the second and third financing plans are intended to facilitate the provision of care at home by making housing a private responsibility, both are considered to be extramural financing plans.

## Methods and Data

We applied a mixed-methods approach in which we combined quantitative and qualitative data to answer our research question.

### Quantitative Methods and Data

#### Definitions

Dutch for-profit nursing homes are defined as facilities that have the legal status of a private for-profit company (private limited company, general partnership, or sole proprietorship). A private equity firm is defined as a company that owns and trades unlisted, private companies; it creates 1 or more funds that obtain capital commitments from investors such as pension funds, insurance companies, or wealthy individuals. Using the fund’s capital, along with a loan commitment, the private company acquires so-called portfolio companies, which are sold within 3 to 7 years on average.

#### Data sources

No available dataset included all the different types of Dutch nursing homes. Hence, we constructed such a dataset for this study based on 2 (semi-) public datasets: data from the Netherlands Patients Federation (ZiN) (2019) for the period 2015–2017 and data from the Dutch National Healthcare Institute of 2016.^33^ We added data on regional characteristics (i.e., socioeconomic indicators) from the Netherlands Institute for Social Research^34^ and Statistics Netherlands.^35^

#### Variables and analysis

To ascertain the legal status, types of ownership, and year of opening for each for-profit nursing home, we searched their respective websites, local news articles (using LexisNexis), ownership information from the Amadeus dataset (financial data and company information for European companies; Bureau van Dijk), and publicly available inspection reports of the Dutch Health and Youth Inspectorate. We then tried to obtain missing data through e-mail correspondence with the nursing homes. We also constructed a dichotomous variable for chain membership; nursing homes were categorized as chain members if they were part of a parent company with 2 or more nursing homes. Furthermore, we calculated the percentage of nursing homes owned by the 4 biggest chains and used the Dutch National Healthcare Institute dataset to estimate the average number of clients living within the different types of nursing homes. The Netherlands Patients Federation data were used to identify significant differences in the client ratings between the nursing home types, conducting the Welch t-test that corrects for unequal variances.

Regional statistics include the socioeconomic status of the region and the average value of the buildings in euros. Regional statistics were linked by means of 4-digit postal codes. The socioeconomic status uses a standardized measure in which zero equals the average Dutch neighborhood and scores are higher (positive) or lower (negative) than the socioeconomic status average.

### Qualitative Methods and Data

In addition to the quantitative analyses, we carried out a qualitative analysis to identify the distinctive features of for-profit nursing homes and to understand the factors that hinder and stimulate the growth of the Dutch for-profit nursing home industry. The research ethics committee exempted this research for the Medical Research Involving Human Subjects Act.

#### Data collection

Twenty-two semi-structured, in-depth interviews were conducted with a total of 25 participants (see Table 1). All participants signed an informed consent form. The interviews consisted of the following 2 questions for directors and experts in the nursing home sector: (A1) What is the organizational model in the for-profit nursing home? (A2) What are opportunities and barriers for growth of the for-profit nursing home industry? Other questions were applied in interviews with the client representatives of for-profit nursing homes: (B1) What were the reasons to choose this particular nursing home? (B2) What were the reasons to choose a for-profit nursing home? (B3) How do you evaluate living in a for-profit nursing home? Interviews were audiotaped and transcribed verbatim.

**Table 1.** Profile of the Participants.

	Interview participants	
Background	*n* = 25	Participant number
Director/staff for-profit facility (facility related to chain)	5	6, 8, 22
Director/staff for-profit facility (standalone facility)	5	5, 10, 11, 12
Client for-profit facility (or representative of a client)	5	1, 2, 3, 14, 15
General sector expert	5	4, 7, 9, 13, 16
Institutional actor^a^	4	17, 18, 19, 21
Director/staff nonprofit facility^b^	1	20

a Participants from the Ministry of Health, long-term care trade association, nonprofit sector, and LTC offices.

b The table lists the current positions of participants, many of whom also had expertise or experience in the nonprofit sector.

#### Sampling

Participants were purposefully selected based on preselected criteria. These included: (a) the participant has expertise on the Dutch nursing home sector, (b) this expertise is based on at least 3 years of experience (this criterion does not apply to the client group of participants), and (c) the expertise was expected to add to the range of perspectives included in the sample. As the study had an explorative basis, maximum variation sampling was applied to capture a wide range of perspectives. We stopped adding new participants to our sample when we reached thematic saturation.

#### Data analysis

We applied inductive thematic techniques to identify major underlying themes in the interview data using Atlas.ti. Two researchers independently drafted a list of recurrent codes derived from the data. The 2 researchers collaboratively refined an initial set of codes that captured the main ideas in the data. Subsequently, the codes were collated into broader themes. For all themes, both the number of coded interview segments on the theme and the number of respondents who shared information on the theme were written down to weigh the relative importance of the themes and to determine the central findings.

## Results

We start by outlining relevant regulatory, political, and cultural context variables. Thereafter, we provide a description of the current makeup of the Dutch nursing home sector, including the distinctive characteristics of for-profit nursing homes. The last paragraphs present our findings on the sector conditions and the broader trends that stimulated the for-profit expansion in the Dutch nursing home industry.

We acknowledge that many factors are strongly interconnected, but we discuss each factor separately for the sake of analytical clarity. The dynamics between the factors are addressed in the Discussion and Conclusions section.

### Context

#### Regulatory context

The LTC reform of 2015 provided 2 opportunities for for-profit entry and expansion in the Dutch nursing home sector.

First, the profit ban for intramural care services prohibits the allocation of profits to third parties for nursing homes that apply the in-kind intramural care package. However, the ban does not apply to care delivered through the extramural financing plans (i.e., HCP, MCP, and PB) or to nursing homes with fewer than 7 people.^36^ Although these extramural plans were introduced to facilitate the provision of care at home, they are increasingly used to provide nursing home care for groups of care-recipients at 1 specific location – that is, the clustered provision of extramural care. In this way, for-profit nursing homes circumvent the ban on profit distribution, but are still able to receive public funding to provide care to people who are assessed by the Care Assessment Centre as requiring nursing home care.

Second, affluent residents of nonprofit nursing homes must make high copayments, and this opened up a market for for-profit nursing homes. All 3 financing plans (in-kind, HCP/MCP, and PB) come with obligatory copayments that vary with residents’ income and capital. The maximum copayment is €2,365 per month for the in-kind intramural package and €862 per month for the extramural financing plans in 2019. This system of obligatory copayments is beneficial for the for-profit sector, as the copayment in their financing plans (HCP/MCP and PB) is much lower than for the in-kind package in nonprofit nursing homes. As a result, the in-kind intramural package is less attractive for more affluent clientele, who can use the €1,500 per month difference in copayments to rent an apartment in a for-profit nursing home. For the majority of for-profit nursing homes, prices for rent and services range from €3,000 to €4,500 per month, but could reach €7,500 per month.^37^ The cost of care, which is covered by public budgets and obligatory copayments, is additional to the monthly rent and services prices (i.e., “topping up” services).^30^

#### Political and cultural context

The Netherlands should be considered a hybrid welfare state, resembling different welfare state types.^38^ The Dutch political context represents a decision-making model that is consensual, decentralized, horizontal, and in collaboration with its stakeholders.^39^ Its political context is characterized by a collaborative relationship between government and nonprofit sectors. Nonprofit enterprises have been the dominant organizational form in the Dutch nursing home sector since World War II.^40^ Capital funds for nonprofit entities were widely accessible and, as a consequence, the entrance of for-profit providers in the health care sector was discouraged.^40^ The preference for nonprofit providers was legally reinforced by a profit ban in 1977.^41^

### Characteristics of the For-Profit Sector

Table 2 provides an overview of the descriptive statistics on the Dutch for-profit nursing home sector in 2019, which consists of 274 for-profit nursing homes, 12.2% of the total number of nursing home locations. For-profit nursing homes are much smaller than their nonprofit counterparts: Whereas for-profit homes have 20 clients on average per location, nonprofit homes average 64 clients per location. This implies that approximately 4.0% of the total nursing home client population lives in for-profit homes.

**Table 2.** Descriptive Statistics For-Profit Nursing Home Sector.

	Nonprofit	For-profit contracted by the regional LTC office (HCP/MCP)	For-profit financed by personal budget
Number of nursing home locations	87.8%n = 1968^a^	12.2%n = 274^b^	
		5.9%	6.3%
		n = 132	n = 142
Average number of clients^c^	64.2 (58.11)n = 1678	22.9 (19.52)n = 32	15.5 (5.13)n = 21
**Legal status ultimate owner**			
Limited liability firm		98.5%	93.0%
Sole proprietorship or general partnership		1.5%	7.0%
**Type of owner**			
Privately owned		53.8%	78.9%
Investor		7.6%	19.0%
Private equity		20.5%	3.5%
International chain		26.5%	0.7%
**Chain affiliation**			
Chain membership	95.2%	81.8%	69.0%
Percentage nursing homes owned by the 4 biggest chains	6.1%	38.6%	40.9%

**Geographical distribution**				
Average SES (2017)^d^	−0.33(1.18)	−0.10**(1.21)	0.13***(1.07)
Average value buildings (×1,000 in euros)^e^	210.54(50.38)	219.88**(61.33)	219.48*(62.87)

Abbreviations: HCP, home-care package; LTC, long-term care; MCP, modular care package; SES, socioeconomic status.Data adapted from Netherlands Patients Federation, National Healthcare Institute (ZiN), Netherlands Institute for Social Research, Statistics Netherlands.

Standard deviation between parentheses.

a The number of intramural care providers in the ZiN dataset.

b Eight for-profit nursing homes were excluded, as it is unknown which financial package they apply; 20 nursing homes were excluded because they work from HCP/MCP, but obtained a nonprofit status.

c Estimation based upon the numerator of the rate of psychotropic drug use per nursing home (ZiN dataset); since not all nursing homes reported on this measure, the number of nursing homes is smaller than the total number of nursing homes.

d Based upon a standardized measure: 0 represents the average Dutch neighborhood.

e In the region of the residence.

*P < .1; **P < .05, ***P < .01.

The majority of for-profit facilities are chain-affiliated. The proportion of for-profit nursing homes that are standalone is higher for homes that rely on PBs than for homes that rely on HCP/MCP. Most for-profit locations are owned by private individuals. One in 5 publicly contracted for-profit nursing homes is private equity-owned; 1 in 4 is owned by an international chain.

Finally, our results show that for-profit nursing homes are more frequently located in affluent regions. For-profit facilities working from a PB, in particular, are situated in regions with a significantly higher socioeconomic status and with a higher average value of buildings.

**Figure 2.**
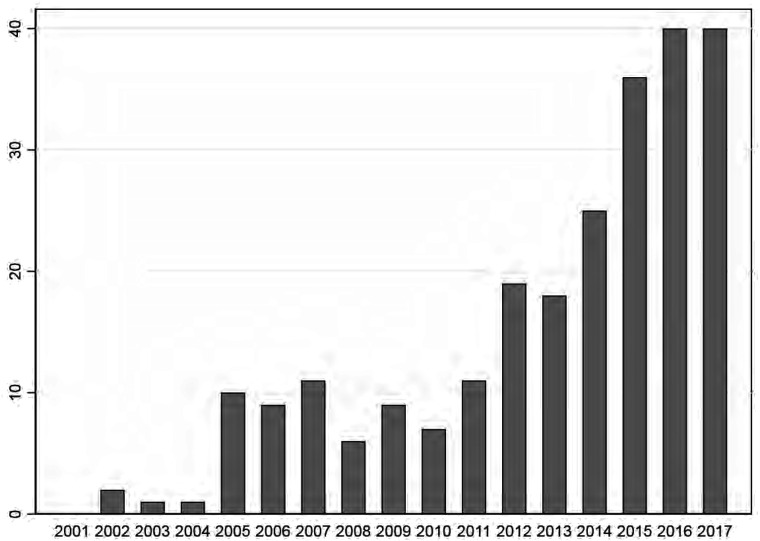
Growth for-profit sector (year of opening). There was 1 missing and 16 locations were opened in 2018, but were already included in the Netherlands Patients Federation dataset of 2017. Facilities that were closed were not included in the dataset.

We found that the for-profit nursing home industry grew substantially over the years: 50% of the active for-profit nursing homes opened in the last 3 years (Figure 2). Approximately 50% of the for-profit nursing homes were already active before the LTC reform of 2015. These for-profit nursing homes relied on private payments or PBs. During our research, we obtained plans of for-profit chains indicating their intentions to open 45 new nursing homes in the near future, implying short-term future growth of at least 16% of the total number of for-profit nursing homes relative to 2017.

We also found an increasing uptake of HCP packages, which reflects the growth of the for-profit nursing home sector. Although HCP packages can be used to fund care at home, respondents highlighted that these packages are primarily used for clients in clustered living facilities that are mainly for-profit. The increase in HCP uptake is much higher (17% in 2016 relative to 2015 and 19% in 2017 relative to 2016) than for in-kind intramural packages (–2% and –1%, respectively).^42^

### Sector Conditions

#### “Cream skimming” clients

For-profit nursing homes exploit the sector’s “cream-skimming” potential by selecting the type of clients they wish to serve. For-profit nursing homes working from the PB plan are able to select their clients, whereas other nursing homes must accept clients referred to them by the LTC office. Participants from the for-profit sector confirm that they select clients based on how they fit with the existing group of residents and on employees’ ability to take care of certain client needs (i.e., severity of their disease). Moreover, despite the promise that clients can live in for-profit facilities until they die, participants mention examples of residents who, because of the severity of their disease, still had to move to a nonprofit nursing home.

#### Inadequate responsiveness

For-profit nursing homes seem more responsive to changing demands than their nonprofit counterparts. There have been increasing shortages in the Dutch nursing home sector; the number of people on waiting lists has almost doubled since 2017.^43^ This left a vacancy for the for-profit sector to fill.

Moreover, for-profit nursing homes have been more responsive to the increased demand for a “well-being” approach that focuses on well-being rather than the medical aspects of nursing home care and that encourages small-scale nursing homes that feel “just like home”. Participants state that for-profit nursing homes are frontrunners in the implementation of the “well-being” approach, whereas the nonprofit sector often represents large-scale, bureaucratic, and medically oriented organizations. The qualitative data further indicate that the elderly of today, and their families, are increasingly demanding: They articulate their wishes and ask for environments that fit their lifestyle, which often does not align with the current supply of traditional nonprofit nursing homes.

Participants provided numerous illustrations of what the “well-being” approach means in practice. For example, the quality of food and food preparation is regarded as an important aspect of well-being. Another aspect of well-being is the living environment of for-profit facilities, which often includes nice outdoor spaces and large private rooms that residents can furnish themselves so that they feel at home, whereas many nonprofit nursing homes provide fully furnished rooms. Client participants stated that they also considered choosing a nonprofit nursing home, but that these looked too much like “institution[s]” (P2) or were “too clinical” (P3). In contrast, for-profit locations have common rooms that “look like a hospital or traditional nursing home as little as possible” (P11) – for example, through “open front doors for residents [with dementia], and the absence of safety measures at the stairs” (P22).

Our tentative analysis of the client ratings of the Netherlands Patients Federation finds that the well-being and customization approach in for-profit nursing homes is highly appreciated by residents. Although the number of for-profit nursing homes in our sample is relatively small, we find that client satisfaction is significantly higher at for-profit providers for all indicators (Table 3).

**Table 3.** Difference Between the Type of Nursing Homes and Their Client Ratings.

	Nonprofit	For-profit^a^
Average score accommodation (scale 1–10)	7.94 (0.58)	8.78*** (0.39)
Average score employees (scale 1–10)	8.16 (0.43)	8.77*** (0.48)
Average score for listening (scale 1–10)	7.78 (0.48)	8.39*** (0.61)
Ratio of clients who would recommend the nursing home (dichotomous variable: yes/no)	0.92 (0.08)	0.95*** (0.07)
N	1.108	32

Data adapted from Netherlands Patients Federation (2014–2017).

Standard deviation in parentheses.^a^All for-profit providers were combined (HCP/MCP- and PB-financed) because the number of observations was deemed too low to separate the 2 groups.

*P < .1; **P < .05; ***P < .01 (alternative hypothesis that for-profit ratings > nonprofit ratings).

Although nonprofit nursing homes aim at moving in the direction of the “well-being” approach and small-scale units, they are hindered by their heritage of large-scale real estate and an organizational culture in which the medical perspective on nursing home care is strongly embedded: “Most for-profit providers benefit from their newness” (P21). The Dutch for-profit nursing homes do not start as large-scale organizations that converted from nonprofit to a for-profit status, but rather function as newly established organizations.

#### Utilizing the current care system

We found another factor that benefitted for-profit nursing homes and does not fall neatly into one of the predefined theoretical categories. Whereas most nonprofit nursing homes employ staff for specialist care, for-profit homes can reduce labor costs by not hiring expensive staff for specialist care. Instead, specialist care in HCP/MCP-funded for-profit facilities often relies on geriatric specialists seconded from nonprofit providers. Specialist care in PB-funded for-profit facilities relies on general practitioners (GPs). Hence, for-profit nursing homes greatly benefit from the wider health care system: They utilize the current care system to reduce their labor costs. GPs have raised their concerns about the limits of their profession in this organizational model:

There was fuss about the role of the GPs in for-profit nursing homes working from PBs. Formally, these elderly live at their own home, which makes the GP the first point of contact for medical care. When 20 elderly people with severe dementia live in one place, however, it can be questioned whether this is manageable for GPs. (P21)

GPs perceive the care for the elderly in these types of homes as too severe and too specialized. Consequently, in 2019, the Dutch Ministry of Health, Welfare and Sport began questioning this for-profit nursing home strategy.^44^

Although participants observed that the “well-being” demand is primarily articulated by more highly educated elderly, our data provide no clear evidence for the heterogeneity of demand proposition as presented in the theoretical framework.

### Broader Trends

#### Demographic

Demographic trends have led to an increase in both the absolute and relative number of elderly in the Netherlands, and this trend is likely to continue in coming decades.^45^ On average, the new generation of elderly is better educated than previous generations and wealthier in terms of equity.^45^ More than half of the elderly in the Netherlands have wealth in excess of 100,000 euros, and 1 in 10 have wealth in excess of half a million euros.^46^ The older population is often able and willing to pay extra for a nice place to live and for extra services. One client participant stated, for example: “I asked my sons: is it financially possible for me to live here? It was no problem. (. . .) Then what else can I wish for?” (P14).

#### Labor market

The qualitative data highlight an important labor market trend: The relative size of the labor force diminishes while nursing homes need extra health care professionals.^45^ Respondents from both the for-profit and the nonprofit sectors stated that labor shortages are to the relative benefit of for-profit nursing homes. The for-profit business model enables more time with clients, as the additional financial income for services is also used to deploy personnel. Moreover, the PB funding plan liberates for-profit nursing homes from several bureaucratic rules by which nursing homes that rely on traditional in-kind funding plans must abide. Participants from the for-profit sector state that they “avoid the red tape that comes from working with LTC offices” (P10); consequently, more time is available to be with clients. Participants also observe more “hospitality employees” at for-profit nursing homes, such as cooks and hostesses: “attention personnel” (P22) who relieve the work of medical staff. As a result, for-profit nursing homes seem to be more attractive employers and face less difficulty in attracting care professionals.

#### Financial

Increasing financial pressure on the Dutch health care system seems to have contributed to the growth of for-profit providers. Without cost-cutting, the health care budget is forecasted to double in 2040, compared to 2015, crowding out financial sources for other collective goods.^47^ The LTC reform of 2015 aimed at bending the increasing cost curve, leading to decreased LTC funding.^32^ After a loud public outcry against the austerity cuts to LTC and its consequences (e.g., care-quality scandals, long waiting lists in nonprofit homes, and the deteriorating reputation of nonprofit nursing homes), LTC received significant extra public funding from 2017 on.^48^ “Elderly do not want to go to a traditional [nonprofit] nursing home; these homes rightly have a bad name.” (P11). Compared to sectors for domiciliary care and care for the disabled, the nursing home sector has been financially weak.^49^ In 2016, 39% of the nursing homes were loss-making entities.^50^ According to the participants, many for-profit firms are less affected by these circumstances, mainly because their revenue model combines private and public funding. Where public funding for care costs (case-mix adjusted annual fees) is tight, the private funding arrangements in the Dutch for-profit nursing home sector allow homes to compensate by increasing fees for real estate and for additional services and amenities.

Another relevant financial trend is the changing access to and costs of financial capital. Due to market-oriented health care reforms, nonprofit health care providers bear more financial risks, which makes banks more reluctant to issue loans.^41^ For-profit nursing homes have easier access to capital because they can circumvent the dependency on bank loans – for example, by turning to private equity firms. Private equity firms can inject large sums of money into the for-profit sector, enabling it to expand quickly. Indeed, we found that private equity firms are active in the for-profit nursing home sector (Table 2). Once their investments have generated growth in the for-profit providers, private equity firms tend to sell the provider. Three private equity-owned Dutch nursing home chains were sold to international chains, comprising 49 locations in total. In all 3 cases, they were sold to French health care chains (Korian or Orphea). Several respondents expressed their concern about private equity firms’ involvement in the for-profit nursing home sector as their focus might be on short-term profit maximization at the expense of quality. Client rating data tentatively suggests lower scores for private equity firm-owned nursing homes than other for-profit entities (Table 4).

**Table 4.** Private Equity Ownership of Nursing Homes in 2016; Client Ratings 2014–2017.

	Non-private equity-owned nursing home	Private equity-owned nursing home
Accommodation	8.84	8.63*
	(0.43)	(0.31)
Employees	8.91	8.46***
	(0.44)	(0.44)
Listening	8.62	8.01***
	(0.50)	(0.55)
Information	8.44	7.88***
	(0.55)	(0.60)
Recommendation	0.97	0.92**
	(0.04)	(0.07)
N	19	16

*P < .1, **P < .05, ***P < .01.

Although participants from the for-profit sector mentioned examples of the use of technology (e.g., home automation), technological trends were not mentioned as a main trend that explains the current for-profit sector expansion.

## Discussion and Conclusions

This study is, to the best of our knowledge, the first academic study aimed at mapping for-profit nursing homes in the Netherlands and understanding the factors that stimulated their growth. We found substantial recent growth in for-profit nursing homes in the Dutch LTC system. Fifty percent of the currently active for-profit homes were established in the last 3 years, resulting in a for-profit market share of 12% (measured in the number of nursing home sites). In comparison to their nonprofit counterparts, Dutch for-profit nursing homes are more often small-scale and more focused on high-income clients. The for-profit sector consists of both standalone homes and chains, including private equity-owned chains.

An interrelated mix of context variables, sector conditions, and broader trends has stimulated for-profit nursing home expansion in the Netherlands. First and foremost, the regulatory context changed. Reforms designed to encourage deinstitutionalization of elderly care unlocked opportunities for the for-profit nursing home sector. For-profit nursing homes embraced new extramural funding plans that allowed them to circumvent the for-profit ban. In other words, the for-profit sector exploited loopholes in the regulatory framework. We found that the peak of the number of newly established for-profit nursing homes coincided with the implementation of LTC reform.

In addition, several sector conditions created opportunities for for-profit newcomers in the nursing home sector. A first condition was the inadequate responsiveness of the dominant nonprofit nursing home sector. The nonprofit sector was unable to respond to the demographically driven increase and change in demands of a new generation of elderly. The for-profit sector provided an alternative to traditional nonprofit nursing homes. For-profit nursing homes were able to acquire this role because most of the for-profit nursing homes are newly established organizations, able to design their organizational models from scratch. For-profit nursing homes established a well-being approach that tallied with the wishes of their clientele, whereas nonprofit nursing homes were less able to do so. This finding runs contrary to findings in Nordic countries (i.e., Denmark, Finland, Norway, and Sweden), for which a previous study found that traditional nursing homes were able to reform their nursing homes from a medical to a social care model.^30^ Tentative analyses find that for-profit providers’ focus on well-being resulted in higher client ratings than the nonprofit sector.

A second sector condition encouraging for-profit sector growth was the “cream skimming” potential for for-profit nursing. We found that for-profit organizations target a relatively affluent clientele, partly in response to the greater wealth of the current generation of elderly compared to previous generations. The PB-financed nursing homes are particularly able to reap the benefits of “cream skimming” because they enjoy more freedom to select their clients than the HCP/MCP-funded, for-profit nursing homes.

A third sector condition was the design of a for-profit business model that relies heavily on the wider care system for specialist care by using geriatric specialists seconded from nonprofit providers or by relying on GPs. For-profit nursing homes reduce labor costs by utilizing the wider health care system. This “system utilization” was not found in literature and therefore adds to our understanding on what factors stimulate the expansion of for-profit providers in mixed markets.

These sector conditions need to be seen in the context of the aforementioned demographic changes, as well as financial and labor market changes. Because of an affluent clientele that pays for additional services and because of their avoidance of red tape in the case of PB-financed care, for-profit nursing homes have more financial leeway to hire “attention staff” and to have a high staff/client ratio. This, in turn, makes for-profit homes more attractive employers relative to nonprofit nursing homes. Hence, labor shortages are to the relative benefit of for-profit nursing homes. In addition, an important financial driver for the for-profit providers’ rise was their access to financial capital from private investors (including private equity firms). The money injection by private equity firms fostered the for-profit sector’s growth, whereas nonprofit organizations were unable to attract such capital and also faced difficulties in getting bank loans. Furthermore, the financing of for-profit organizations with both public and private funding enabled them to rely less on public funding, shielding them somewhat from austerity measures.

### Limitations

Our methods come with some limitations. First, specific case-mix control variables were not available. Our qualitative data indicate that nonprofit nursing homes tend to have a heavier case mix, but this could not be controlled for in our study. Second, our view of for-profit nursing homes is limited to homes detected by the Netherlands Patient Federation. Since some standalone homes might be unknown to them, there might be a slight underreporting of the number of for-profit homes. Third, a relatively low number of for-profit nursing homes received 15 or more client ratings in the Netherlands Patients Federation dataset. We therefore present these quantitative data as supporting evidence to our qualitative findings. Finally, a large proportion of the participants in our study were working in or affiliated with the for-profit sector, which might lead to a bias in the qualitative data in favor of for-profit nursing homes. Data from the for-profit sector were therefore constantly compared to data from other participants. Results were only included if they were confirmed by participants from different backgrounds (Table 1).

### Implications

The growing for-profit nursing home sector sparks governance questions. Based on the qualitative and quantitative findings, we outline several possible governance implications related to the composition of the market, care-quality norms, and accessibility.

For-profit nursing home growth has 2 interconnected implications for the market composition of the Dutch nursing home sector. The first relates to market consolidation. The 4 biggest chains in the for-profit sector in the Netherlands already own about 40% of all for-profit nursing homes. Consolidation could have negative consequences for the quality of care: Studies on U.S. nursing homes have found that for-profit nursing home chains provide inferior quality of care.^51,52^ The second implication relates to private equity firms investing in for-profit nursing homes. In countries such as Sweden, Norway, Canada, the United Kingdom, and the United States, private equity firms are active within the nursing home sector.^6,53^ Our data show that Dutch nursing home chains are also partly owned by these firms. The consequences are unclear because the international evidence on the quality performance of private equity firms is inconsistent: Studies present both indications of lower quality in private equity homes^51,54^ and no harm to quality of care.^55^ Our data tentatively suggest that client ratings are lower among private equity-owned nursing homes (Table 4). The changing composition of the Dutch nursing home sector toward for-profit chains and the presence of private equity firms demands close scrutiny with regard to their long-term consequences.

A second and related implication of the presence of the for-profit sector concerns quality norms. We found that for-profit nursing homes seem to score better on client satisfaction rates – in contrast to U.S. findings,^56^ but in line with findings from Sweden.^29^ The latter study reported that private nursing homes “seem to focus more on personal service aspects rather than on structural prerequisites for care quality.”^29(p565)^ Most literature reviews from the United States report lower care quality in for-profit nursing homes than in nonprofit homes.^2–4^ Studies in Nordic countries do not unequivocally support these findings.^5,6^ Further research is needed on how for-profit ownership affects care quality in Dutch nursing homes.

Last, the presence of the for-profit sector also has implications for the accessibility of the nursing home sector. Although we found some examples of for-profit nursing homes that target low- and middle-income groups, the majority of for-profit nursing homes target high-income elderly. The “cream skimming” behavior of for-profit providers further perpetuates the polarization of the nursing home sector. These 2 factors raise concerns about the general accessibility of the Dutch nursing home system for lower-income groups due to the more limited options available to them and to potential differences in waiting lists.^57^

Although the for-profit sector has possibly eased waiting lists for nursing home care and shaken up the relatively unresponsive traditional LTC market, there are serious governance risks associated with the for-profit sector providing nursing home services. If the for-profit nursing home sector maintains its low profile, as it has been able to do for most of its existence, the societal implications could be profound and might counter the benefits associated with the for-profit sector.
